# Post-migration Stressors and Subjective Well-Being in Adult Syrian Refugees Resettled in Sweden: A Gender Perspective

**DOI:** 10.3389/fpubh.2021.717353

**Published:** 2021-09-09

**Authors:** Nissen Alexander, Sengoelge Mathilde, Solberg Øivind

**Affiliations:** ^1^Department of Health Sciences, The Swedish Red Cross University College, Huddinge, Sweden; ^2^Division for Forced Migration and Refugee Health, Norwegian Centre for Violence and Traumatic Stress Studies, Oslo, Norway; ^3^Department of Global Public Health, Karolinska Institutet, Stockholm, Sweden

**Keywords:** post-migration stressors, gender, subjective well-being, refugee, social support, WHO-5 well-being index

## Abstract

A number of post-migration stressors have been shown to adversely affect mental health in refugees resettled in high-income countries, including poor social integration, financial difficulties and discrimination, and recent evidence suggests that these effects are gender specific. Social support has been found to buffer against post-migration stress in some studies on refugee populations, though the evidence on this is mixed. The present study used cross-sectional survey data from a nationwide, randomly sampled group of adult refugees from Syria resettled in Sweden between 2008 and 2013 (*N*_sample_ = 4,000, *n*_respondents_ = 1,215, response rate 30.4%) to investigate gender-specific associations between post-migration stressors and subjective well-being (SWB) and whether these associations were modified by social support. SWB was measured with the WHO-5 Well-being Index (scaled 0–100), dichotomized into high (≥50) and low (<50) SWB. Main analyses were stratified by gender, and regressed SWB on four domains of post-migration stress (financial strain, social strain, competency strain and discrimination) using logistic regression, adjusting for sociodemographic variables and traumatic experiences. Social support was tested as an effect modifier. In fully adjusted models, main risk factors for low SWB were high financial strain, especially in males (OR_high vs. low strain, males_ = 10.30 [4.91–21.6], *p* < 0.001 vs. OR_high vs. low strain, females_= 3.84 [1.68–8.79], *p* = 0.002), and high social strain, *only* in males (OR_high vs. low strain, males_ = 9.21 [3.96–21.4], *p* < 0.001 vs. OR_high vs. low strain, females_ = 1.03 [0.40–2.64], *p* = ns). There was some evidence that social support buffered the adverse association of financial strain with SWB. In conclusion, the present study found clear support of gender-specific effects of post-migration stressors on SWB. Mitigation strategies and interventions should be aware of and sensitive to these potential gendered effects, and future research exploring mental health in the context of resettlement stress should have a heightened focus on the important role of gender.

## Introduction

Due to the rapidly growing number of refugees from 2015 onwards, there is an urgency and considerable debate regarding durable solutions to meet the psychosocial needs of the 26 million refugees worldwide (1, 2). Currently, 15% of the world's refugees are resettled in a high-income host country (3). Refugees from Syria continue to be the largest forcibly displaced population globally with 6.6 million refugees and more than six million internally displaced people (3, 4), and Syrians have accounted for a substantial part of all asylum applications to the European Union in recent years (5). A large number of studies have documented elevated rates of mental health problems in refugee populations (6–8), and the links between mental ill health and pre- and peri-migration potentially traumatic experiences (PTEs) such as sustained conflict, fleeing under life-threatening conditions, separation from family and friends, and prolonged stays in unsafe and overcrowded camps are well-established (6, 9).

Increasing attention has been given to stressors in the post-migration phase and their potential adverse effects on mental health in refugee populations (10–12). Important post-migration stressors include perceived discrimination, lack of economic opportunity, lack of access to resources, social isolation, family concerns and poor language proficiency. These stressors have been shown to not only have strong negative direct effects on mental health, but also to mediate and moderate the adverse effects of other stressors associated with the refugee experience, including pre- and peri-migration PTEs (13–15). Moreover, a recent longitudinal study on refugees in Australia suggests that the effects of post-migration stressors on mental health change over time, and that these time-changing effects follow different trajectories for males and females (16). The review concludes that studies to date have not adequately focused on and explored gender differences when investigating resettlement stress and mental health in refugee populations. This conclusion is supported by an earlier cross-sectional study on Afghan refugees resettled in the United States which explored gender-specific effects of several post-migration stressors on distress (17). Specifically, the study found that strong family ties as well as English proficiency were associated with lower distress in females, but not males; that acculturation dissonance was associated with higher distress in males, but not females; and that gender ideology and distress were gender specific, with traditional women and egalitarian men experiencing less distress. The latter points to the centrality of gender roles when trying to interpret gender-specific effects of resettlement stress on mental health, and the complex and often challenging processes that influence and shape gender roles in migrating populations relocating from societies and cultures with fairly marked gender separation, to societies where this separation is much smaller (18, 19). Further studies with a gender-focused approach are warranted.

The majority of research on mental health in refugee populations have used a deficit model of health (20–22), though a growing number of studies have started to conceptualize and study *positive* mental health using the overlapping concepts of subjective well-being (SWB), life satisfaction and quality of life (QoL) (23–26), with the latter being the most frequently studied concept. Findings from studies on QoL in relation to post-migration stress have yielded mixed results, with some studies failing to find a link between the two (27), whereas others finding QoL to be adversely associated with living difficulties (28, 29), discrimination (28), and unemployment (30, 31). Interestingly, associations between gender and QoL were found in some studies (32–34) but not others (27, 28), and no study that we are aware of have explored gender-specific associations between post-migration stressors and QoL. However, gender was explored as an effect modifier between perceived discrimination and SWB in a study on refugees in Canada, with evidence of an adverse association for men only (24).

Social support has repeatedly been shown to be protective of mental health in refugee populations (23, 35), and part of this may be related to the potential stress buffering effects of social support. Specifically, one study on Iraqi migrants resettled in Sweden found that social participation and trust in others modified the negative effects of discrimination, housing problems, and financial difficulties on mental health (36), and social support was identified as an important moderator of the relationship between resettlement stress and depression in the Canadian Refugee Resettlement Project (37). In contrast, a study on Afghan refugee in the US which tested whether social support modified the association between perceived discrimination and psychological distress, did not find any evidence to support this (38). Refugee women in particular have acknowledged the importance of social support in adapting to their lives in the host country and report that they face barriers to maintaining it in the resettlement phase, both within their own ethnic community and in the host community, often due to language barriers (39, 40).

A weakness in the field of refugee mental health research to date is the limited focus on and reporting of gender specific findings (7, 16, 17, 40, 41). Based on the mounting evidence of the importance of gender in understanding the complex links between pre- and post-migration stressors and mental health (16, 17), there is a clear need for studies with a gender-focused approach. Therefore, the purpose of the present study was to build on previous findings by our group on the adverse association between post-migration stressors and mental health (14, 42) by applying a gender perspective and positive mental health approach. Specifically, the study's main objectives were to explore and report gender-specific associations of post-migration stressors with subjective well-being (SWB) in adult refugees from Syria resettled in Sweden and evaluate the statistical evidence for gendered effects. A secondary objective was to test whether social support modified the associations of post-migration stressors with SWB.

## Materials and Methods

### Participants

Eligible participants for the present study included all refugees from Syria between 18 and 64 years of age who were granted permanent residency in Sweden on grounds of asylum and resettled in a municipality between 2011 and 2013. A sampling frame of 9,662 eligible individuals was identified through the Swedish Total Population Register (TPR) and a simple random sample of 4,000 individuals was drawn in February 2016. Selected participants were sent a study invitation letter and the Arabic questionnaire *via* postal mail. Of the 4,000 invited individuals, 1,215 returned the questionnaire, giving a response rate of 30.4%. Supplementary Table 2 compare respondents to non-respondents on key sociodemographic variables. For further details on design, setting and participants, we refer to a prior publication by our group (14).

The study was approved by the Stockholm Regional Ethical Review Board (number: 2015/1463-1431 and 2016/549-32).

### Measures

#### Subjective Well-Being

Subjective well-being (SWB) was measured using the WHO-5 Well-being Index (WHO-5), one of the most widely used questionnaires assessing subjective SWB. The questionnaire has shown good psychometric properties across various populations and cultures, including refugees (43–45). The WHO-5 consists of the following five items: (1) “I have felt cheerful and in good spirits,” (2) ‘I have felt calm and relaxed,” (3) “I have felt active and vigorous,” (4) “I woke up feeling fresh and rested,” and (5) “My daily life has been filled with things that interest me.” Respondents are asked to rate how well each statement applies to him or her for the last 14 days using a 6-point Likert scale going from 0 = none of the time to 5 = all of the time. Individual items were summed and multiplied by 4 to create an overall score of SWB ranging from 0 (lowest possible SWB) to 100 (highest possible SWB). A cut-off score of 50 was used to create a dichotomous version over SWB, with ≥50 indicating high well-being vs. <50 indicating low well-being (43). Cronbach's alpha for the scale was 0.94.

#### Post-migration Stress

Four domains from the Refugee Post-Migration Stress Scale, RPMS, focusing on stressors related to the host country, were used to measure post-migration stress: (1) *financial strain*; (2) *social strain*; (3) *competency strain*; and (4) *perceived discrimination*. The RPMS scale was recently developed with the aim of providing an updated instrument for assessing post-migration stress in refugee populations, and the scale has shown good validity when tested among refugees from Syria (42). Please refer to Malm et al. (42) for further details on domain identification and validation. *Financial strain* consists of three items tapping into material and economic hardship potentially threatening integrity, independence, dignity and well-being (example: “Worry about unstable financial situation”). *Social strain* consists of three items relating to social hardship such as feeling isolated or frustrated due to loss of status (example: “Feeling excluded or isolated in the Swedish society”). *Competency strain*, also comprising three items, taps into feelings of inadequacy of host-country specific skills needed to successfully navigate day-to-day living (example: “Bothering difficulties communicating in Swedish”). *Perceived discrimination* consists of four items relating to the experience of unfair treatment in Sweden, either verbally or nonverbally, on the basis of prejudice (example: “Feeling disrespected due to my national background”). Respondents are requested to indicate how frequently they experience the different items on a scale ranging 1 = never to 5 = very often. Respondents were split into three groups for each domain: low, medium and high strain. The low-strain group consisted of respondents with a maximum score of 2 = seldom for all items within a given domain. The high-strain group consisted of respondents who answered 4 = often or 5 = very often on all items within a given domain, and the medium-strain group consisted of everyone else. Cronbach's alpha for the four domains ranged between 0.80 and 0.84.

#### Social Support

Social support was assessed using the ENRICHD Social Support Instrument, with Items 4 (“Is there someone available to help with daily chores?”) and 7 (“Are you currently married or living with a partner?”) excluded (46). The remaining 5 items (e.g., “Is there someone you can count on to listen to you when you need to talk?”) measure instrumental and emotional support and are all scored on a 5-point Likert scale going from 1 = none of the time (i.e., low social support) to 5 = all of the time (high social support). The scale has previously been tested among refugees from Syria and found to have adequate psychometric properties in this population (47). Items were summed to create a total score ranging from 5 (lowest possible social support) to 25 (highest possible social support) and a cut-off point of 18 was used to create a dichotomized version of the scale, with ≤ 18 =low social support vs. >18 = high social support.

#### Pre- and Peri-Migration Potentially Traumatic Experiences

Potentially traumatic experiences (PTEs) were measured with the Refugee Trauma History Checklist (RTHC), which has been tested and validated in a sample of Syrian asylum seekers in Sweden (48). The scale asks about eight PTEs (e.g., “War at close quarter” and “Forced separation from family or close friends”) with reference to the time-periods *before* (pre-flight) or *during* (peri-flight) migration, for a total of 16 PTE items. To enhance comparability with prior evidence, participants were categorized based on a calculated *PTE adversity ratio* (PTE-AR) introduced by Steel et al. in their review in 2009 (6). Specifically, the PTE-AR is estimated as the number of endorsed PTEs divided by the total number of PTEs inquired about (i.e., 16 in the present study), and categorized into: < 0.2; 0.2–0.29; 0.3–0.39; and ≥0.4.

#### Sociodemographic and Background Variables

Gender was an integral part of the study's main objective and based on registry data from the Swedish Total Population Register (TPR). Other sociodemographic variables included: age (18–29, 30–39, 40–49, and ≥50 years); years of education (0–9, 10–12, 13–14, and ≥15); civil status (married, unmarried, and divorced/separated/widowed); and year of immigration (2008–2011, 2012, and 2013). Data for all variables were obtained through the TPR as well.

#### Psychological Distress

Psychological distress was included in sensitivity analyses in order to evaluate potential confounding by psychological distress of the main associations of interest. Symptoms of anxiety and depression were measured with the Hopkins Symptom Checklist, HSCL-25 (49), and the first 16 items from section Discussion of the Harvard Trauma Questionnaire (HTQ) were used to measure symptoms of PTSD (50). Both scales have been applied extensively in studies on refugee mental health in the last decades (51). All three variables (i.e., anxiety, depression and PTSD) were included in dichotomized form in the sensitivity analyses based on whether mean item scores were above threshold values suggesting checklist-positive disorder. Specifically, mean item scores above 1.75 (first 10 HSCL items) and 1.80 (last 15 HSCL items) were used to define checklist-positive anxiety and depression, respectively, and a mean item score above 2.06 was used to define a checklist-positive PTSD. In order to underline that these mental health variables are based on symptom checklist scores and not the gold standard clinical interview, we use the prefix “checklist-positive” or refer to “symptoms of.”

### Statistical Analysis

Data were first checked for outliers, errors and missing using simple frequency distributions and cross tabulations. The total number of participants with missing data for a given variable and the total number contributing data to full multivariable models are indicated in the tables. The outcome variable, the WHO-5 well-being index, was summarized across predictors and background variables using both the mean score with standard deviation (SD) and the proportion in the high (≥50) and low (<50) SWB categories.

Associations between SWB and predictors/background variables were tested using chi-square test for categorical variables as well as crude and adjusted logistic regression analysis. The logistic models compared the odds of low vs. high SWB across predictor levels, summarized as odds ratios (ORs) with 95% confidence intervals (95% CIs) against a set reference. Sociodemographic variables were included in the full regression models for a priori reasons based on theory and prior evidence of importance, regardless of whether they showed independent effects. As exposure to potentially traumatic experiences (PTEs) has repeatedly been shown to be closely linked to mental health and well-being, which could subsequently affect the experience of post-migration stressors, PTE-AR was included in the final model to adjust for this potential distortion of the true and direct effects of post-migration stressors on SWB. All four post-migration stressors were included in the final models, though stepwise exploration of each stressor's association with SWB adjusting for demographic variables and PTE-AR was also done. As a sensitivity analysis, we repeated the final regression models with symptoms of psychological distress included in the models (i.e., checklist-positive anxiety, depression and PTSD) to evaluate potential confounding by these variables. Multicollinearity was checked before running full models.

To investigate gender-specific associations between post-migration stressors and SWB, analyses were stratified by gender and gender was tested as an effect modifier by sequentially fitting interaction terms between gender and each post-migration stressor. That is, interaction between gender and one post-migration stressor was tested at a time, first in crude models, then in fully adjusted models. The presented *p*-values for interaction are based on Wald test of H_0_ = no interaction (i.e., uniform effect across gender). To determine whether interaction was present, the magnitude of strata-specific effects, confidence intervals, sensitivity analysis and *p*-values from the Wald tests for interaction were evaluated together, with a significance threshold for the latter test not fixed at < 0.05 (52). Social support was subsequently tested as an effect modifier between post-migration stressors and SWB for stressors which showed significant associations with SWB. This was done in the same manner as for gender.

Because there was a fairly high number of participants with missing data in the final regression models with complete-case analysis, sensitivity analyses for these models were done with imputed data obtained through multiple imputation by chained equations, MICE (53). The imputation model contained all variables in the final analysis model and imputed males and females separately to account for gender interactions in the models.

All statistical analysis were conducted with Stata, version 16 (STATA Corporation, College Station, TX, USA).

## Results

Descriptive statistics on study participants as well as summary statistics on SWB across predictors and background variables are presented in Tables 1, 2. For the total sample, the mean SWB score was 57.7 (standard deviation, SD = 27.1, *n* = 1,173) and 725 (61.8%) of participants were categorized as having high SWB vs. 448 (38.2%) as low. All post-migration stressors, gender, education and potentially traumatic experiences (PTE-AR) were associated with SWB in bivariate analyses.

**Table 1:** Descriptive statistics on background variables and post-migration stressors; bivariate association with gender.

		**All respondents**		**Stratified by gender**				
				**Males**		**Females**		**χ^**2**^**
		***n***	**(%)**	***n***	**(%)**	***n***	**(%)**	
Age								0.13
	18–29	283	(23.29)	161	(21.10)	122	(26.99)	
	30–39	400	(32.92)	257	(33.68)	143	(31.64)	
	40–49	295	(24.28)	193	(25.29)	102	(22.57)	
	≥50	237	(19.51)	152	(19.92)	85	(18.81)	
Education								0.04
	0–9 yrs	453	(38.42)	293	(39.43)	160	(36.70)	
	10–12 yrs	255	(21.63)	169	(22.75)	86	(19.72)	
	13–14 yrs	234	(19.85)	129	(17.36)	105	(24.08)	
	≥15 yrs	237	(20.10)	152	(20.46)	85	(19.50)	
Civil status								<0.001
	Married	771	(63.46)	439	(57.54)	332	(73.45)	
	Unmarried	386	(31.77)	301	(39.45)	85	(18.81)	
	Divorced/wid.	58	(4.77)	23	(3.01)	35	(7.74)	
Immigration year								0.13
	2008–2011	76	(6.27)	54	(7.11)	22	(4.87)	
	2012	334	(27.56)	217	(28.55)	117	(25.88)	
	2013	802	(66.17)	489	(64.34)	313	(69.25)	
PTE-AR								<0.001
	<0.20	279	(24.41)	153	(21.46)	126	(29.30)	
	0.20–0.29	128	(11.20)	72	(10.10)	56	(13.02)	
	0.30–0.39	250	(21.87)	149	(20.90)	101	(23.49)	
	≥0.40	486	(42.52)	339	(47.55)	147	(34.19)	
Financial strain								0.26
	Low	284	(23.83)	189	(25.34)	95	(21.30)	
	Medium	692	(58.05)	427	(57.24)	265	(59.42)	
	High	216	(18.12)	130	(17.43)	86	(19.28)	
Social strain								0.87
	Low	326	(27.82)	202	(27.45)	124	(28.44)	
	Medium	713	(60.84)	452	(61.41)	261	(59.86)	
	High	133	(11.35)	82	(11.14)	51	(11.70)	
Competency strain								0.15
	Low	286	(24.22)	189	(25.47)	97	(22.10)	
	Medium	787	(66.64)	493	(66.44)	294	(66.97)	
	High	108	(9.14)	60	(8.09)	48	(10.93)	
Discrimination								0.70
	Low	748	(63.39)	463	(62.57)	285	(64.77)	
	Medium	425	(36.02)	273	(36.89)	152	(34.55)	
	High	7	(0.59)	4	(0.54)	3	(0.68)	
Social support								0.06
	High	524	(44.37)	313	(42.24)	211	(47.95)	
	Low	657	(55.63)	428	(57.76)	229	(52.05)	

*PTE-AR, potentially traumatic experiences adversity ratio; wid, widowed*.

**Table 2:** Summary of WHO-5 Well-Being Index (0-100), mean (SD) and dichotomized into high vs. low subjective well-being (SWB).

		**Mean index score (SD)**		**High (≥50)**		**Low (<50)**		
				***n***	**(%)**	***n***	**(%)**	**χ^**2**^**
Gender								0.008
	Male	59.40	(27.42)	475	(65.52)	259	(57.81)	
	Female	54.82	(26.38)	250	(34.48)	189	(42.19)	
Age								0.177
	18–29	58.78	(26.49)	175	(24.14)	101	(22.54)	
	30–39	59.24	(27.28)	240	(33.10)	145	(32.37)	
	40–49	58.16	(27.40)	185	(25.52)	102	(22.77)	
	≥50	53.08	(26.91)	125	(17.24)	100	(22.32)	
Education								0.031
	0–9 yrs	59.69	(27.88)	281	(40.20)	156	(35.45)	
	10–12 yrs	58.72	(27.75)	162	(23.18)	85	(19.32)	
	13–14 yrs	54.51	(27.12)	122	(17.45)	101	(22.95)	
	≥15 yrs	54.98	(25.16)	134	(19.17)	98	(22.27)	
Civil status								0.139
	Married	58.73	(26.69)	474	(65.38)	272	(60.71)	
	Unmarried	56.93	(27.92)	222	(30.62)	149	(33.26)	
	Divorced/wid.	48.79	(25.97)	29	(4.00)	27	(6.03)	
Immigration year								0.754
	2008–2011	56.45	(28.33)	43	(5.94)	28	(6.28)	
	2012	58.23	(28.59)	206	(28.45)	118	(26.46)	
	2013	57.65	(26.34)	475	(65.61)	300	(67.26)	
PTE-AR								<0.001
	<0.20	65.22	(24.66)	202	(29.40)	73	(17.38)	
	0.20–0.29	59.61	(25.18)	79	(11.50)	45	(10.71)	
	0.30–0.39	58.97	(25.03)	153	(22.27)	87	(20.71)	
	≥0.40	52.27	(28.58)	253	(36.83)	215	(51.19)	
Financial strain								<0.001
	Low	75.14	(19.56)	242	(33.94)	36	(8.13)	
	Medium	56.33	(25.81)	404	(56.66)	267	(60.27)	
	High	38.63	(25.17)	67	(9.40)	140	(31.60)	
Social strain								<0.001
	Low	71.69	(23.92)	266	(37.89)	53	(12.21)	
	Medium	55.19	(24.88)	398	(56.70)	295	(67.97)	
	High	36.71	(27.94)	38	(5.41)	86	(19.82)	
Competency strain								<0.001
	Low	68.27	(24.63)	211	(29.80)	69	(15.86)	
	Medium	55.85	(26.70)	452	(63.84)	309	(71.03)	
	High	45.69	(26.71)	45	(6.36)	57	(13.10)	
Discrimination								<0.001
	Low	61.08	(26.87)	488	(68.64)	239	(55.45)	
	Medium	52.86	(26.01)	221	(31.08)	187	(43.39)	
	High	25.71	(36.06)	2	(0.28)	5	(1.16)	
Social support								<0.001
	High	65.74	(23.61)	389	(54.87)	128	(29.09)	
	Low	50.89	(27.74)	320	(45.13)	312	(70.91)	

*SD, standard deviation; PTE-AR, potentially traumatic experiences adversity ratio; wid, widowed*.

Unadjusted and adjusted logistic models of SWB regressed on all post-migration stressors are presented in Table 3, with the latter two models stratified by gender to investigate gender-specific associations and evidence of interaction. Because there were too few individuals with data in the highest category for discrimination, results for this category were not estimated. There was very strong evidence that financial strain was associated with increased odds of low SWB in the fully adjusted model, and some evidence that this association was stronger in males (OR_high vs. low financial strain, males_ = 10.30, 95% CI 4.91–21.6, *p* < 0.001) compared to females (OR_high vs. low financial strain, females_ = 3.84, 95% CI 1.68–8.79, *p* = 0.001). The evidence for interaction was notably stronger in sensitivity analysis done with imputed data (*p*-value interaction = 0.026) vs. with complete-case data (*p*-value interaction = 0.156), though the changes in ORs were modest. In females, SWB did not differ between the medium and low strain groups (neither in complete-case analysis nor in sensitivity analysis with imputed data), though there was very strong evidence for a difference between these groups in males (OR_medium vs. low financial strain, males_ = 3.59, 95% CI 1.95–6.60, *p* < 0.001), which was even more marked in sensitivity analysis on imputed data (OR_medium vs. low financial strain, males_ = 4.24, 95% CI 2.37–7.57, *p* < 0.001). After adding symptoms of anxiety, depression and PTSD in further sensitivity analysis, SWB was no longer association with financial strain in females, but there was strong evidence for an association in males, albeit with notably reduced ORs (OR_high vs. low financial strain, males_ = 4.67, 95% CI 2.04–10.7, *p* < 0.001, not shown in tables).

**Table 3:** Logistic models of low subjective well-being (SWB) regressed on post-migratory stressors.

		**Total sample (UOR)**		**Gender-specific models (UOR)**					**Gender-specific models (AOR**^**†**^)				
				**Male**		**Female**			**Male (** ***n*** **=** **609)**		**Female (** ***n*** **=** **362)**		
		**OR**	**95% CI**	**OR**	**95% CI**	**OR**	**95% CI**	**Wald^**‡**^**	**OR**	**95% CI**	**OR**	**95% CI**	**Wald^**‡**^**
Financial strain	Low	Ref		Ref		Ref		0.067	Ref		Ref		0.156
	Medium	4.44^c^	(3.03–6.51)	6.45^c^	(3.72–11.2)	2.72^c^	(1.56–4.73)		3.59^c^	(1.95–6.60)	1.66	(0.84–3.27)	
	High	14.05^c^	(8.91–22.1)	21.51^c^	(11.4–40.6)	7.89^c^	(4.0–15.6)		10.30^c^	(4.91–21.6)	3.84^b^	(1.68–8.79)	
Social strain	Low	Ref		Ref		Ref		<0.001	Ref		Ref		0.001
	Medium	3.72^c^	(2.67–5.18)	6.78^c^	(4.03–11.4)	2.12^b^	(1.34–3.37)		4.25^c^	(2.31–7.82)	1.06	(0.58–1.92)	
	High	11.36^c^	(7.01–18.4)	26.04^c^	(13.0–52.4)	4.53^c^	(2.23–9.22)		9.21^c^	(3.96–21.4)	1.03	(0.40–2.64)	
Competency strain	Low	Ref		Ref		Ref		0.452	Ref		Ref		0.325
	Medium	2.09^c^	(1.54–2.84)	2.27^c^	(1.52–3.38)	1.81^b^	(1.11–2.95)		1.32	(0.81–2.17)	0.79	(0.42–1.51)	
	High	3.87^c^	(2.41–6.24)	4.91^c^	(2.62–9.20)	2.64^b^	(1.27–5.50)		2.10	(0.92–4.79)	0.93	(0.36–2.39)	
Discrimination	Low	Ref		Ref		Ref			Ref		Ref		…
	Medium	1.73^c^	(1.35–2.22)	1.90^c^	(1.38–2.61)	1.53^a^	(1.02–2.29)		1.39	(0.94–2.07)	1.21	(0.73–2.02)	
	High	…	…	…	…	…	…		…	…	…	…	

*Low psychological well-being defined as WHO-5 Well-Being Index score <50; UOR, unadjusted odds ratio; AOR, adjusted odds ratio.*

† *Adjusted for age, education, civil status, year immigration, potentially traumatic experiences (PTE-AR), social support, and other variables in the table.*

‡ *p-values for Wald test of interaction between gender and post-migratory stressors (interaction terms were tested sequentially—i.e., only one interaction term were included in each model)*.

There was very strong evidence of gender-specific associations between social strain and SWB (Wald *p*-value for interaction < 0.001), with fully adjusted models showing clear associations in males, but none in females. These findings were very similar in sensitivity analyses done on imputed data. In males, both the medium and high strain group had increased odds of low SWB compared to the low strain group (OR_medium vs. low social strain, males_ = 4.25, 95% CI 2.31–7.82, *p* < 0.001 and OR_high vs. low social strain, males_ = 9.21, 95% CI 4.91–21.6, *p* < 0.001), with ORs pushed slightly toward the null (<5%) in sensitivity analysis on imputed data. The statistical evidence remained strong after controlling for symptoms of anxiety, depression and PTSD, even though the ORs were reduced (OR_medium vs. low social strain, males_ = 3.62, 95% CI 1.84–7.13, *p* < 0.001, OR_high vs. low social strain, males_ = 6.56, 95% CI 2.58–16.6, *p* < 0.001, not shown in tables).

No associations were found between competency strain and SWB or between discrimination and SWB in fully adjusted models using complete-case analysis, and there was only weak evidence (*p* = 0.045) that the high competency strain group had elevated odds of low SWB compared to the low strain group in sensitivity analysis with imputed data.

There was some evidence (Table 4) that social support modified the association between the financial strain and SWB (Wald *p*-value = 0.08), with a stronger association found in the low social support group (OR_high vs. low financial strain, low social support_ = 11.30, 95% CI 5.13–24.9, *p* < 0.001) compared to the high social support group (OR_high vs. low financial strain, high social support_ = 4.33, 95% CI 1.95–9.60, *p* < 0.001). The evidence for interaction was marginally stronger in sensitivity analysis done on imputed data (*p*-value for interaction = 0.07), but the ORs were more or less unchanged, except for the high strain low support group which was attenuated (from OR = 11.3 to OR = 9.21). When analysis was stratified by gender, no evidence of interaction was found. The test for interaction between social strain and social support is not reported because there were ≤ 5 participants in individual cells after stratifying by social support, resulting in unstable parameter estimates.

**Table 4:** Fully adjusted logistic models of low subjective well-being (SWB) regressed on financial strain and testing of social support as effect modifier.

	**Total sample (** ***n*** **=** **974)**					**Gender-specific**									
						**Male (** ***n*** **=** **609)**					**Female (** ***n*** **=** **362)**				
	**High support**		**Low support**			**High support**		**Low support**			**High support**		**Low support**		
	**OR**	**95% CI**	**OR**	**95% CI**	**Wald^**†**^**	**OR**	**95% CI**	**OR**	**95% CI**	**Wald^**†**^**	**OR**	**95% CI**	**OR**	**95% CI**	**Wald^**†**^**
Financial strain															
Low	Ref		Ref		0.08	Ref		Ref		0.49	Ref		Ref		0.36
Medium	1.59	(0.87–2.91)	4.42^c^	(2.24–8.71)		2.11	(0.83–5.37)	4.34^c^	(1.85–10.1)		1.50	(0.64–3.51)	4.36^a^	(1.26–15.1)	
High	4.33^c^	(1.95–9.60)	11.30^c^	(5.13–24.9)		5.66^b^	(1.71–18.7)	12.6^c^	(4.59–34.8)		4.37^a^	(1.38–13.9)	10.8^c^	(2.67–43.5)	
Social strain^*^															

*Low psychological well-being defined as WHO-5 Well-Being Index score <50. Models adjusted for age, education, civil status, year immigration, potentially traumatic experiences adversity ratio, and other post-migratory stressors.*

† *p-values for Wald test of interaction between social support and financial strain.*

* *Interaction between social strain and social support is not reported because there were ≤ 5 individuals in individual cells after stratifying by social support, leading to unstable parameter estimates*.

## Discussion

### Main Findings

To our knowledge, the present study is one of the first to investigate gender-specific associations between post-migration stressors and positive mental health in a population of adult refugees resettled in a high-income country. Our results suggest that post-resettlement social strain in particular, but also financial strain, had clear gendered effects on SWB, with much stronger effects in males than in females. In fact, in fully adjusted analysis, social strain showed no association with SWB in females whereas in males, high social strain was associated with a manyfold increase in the odds of low SWB. An important secondary finding was that social support appeared to buffer the adverse effects of financial strain on SWB.

The present study's moderate evidence for gender-specific associations between financial strain and SWB is somewhat contrary to the study by Wu et al. on refugees resettled in Australia (16), which did not find that the adverse associations of economic stressors with mental health varied by gender. A possible explanation for this could be that the gendered effect only comes into play when mental health is framed and measured in terms of positive mental health and not when focusing on mental health as psychological distress as was done by Wu et al. Another possible explanation could be that “financial strain” as operationalized in our study measured a somewhat different and narrower construct than the “economic stressors” variable in the Wu et al. study. Specifically, our study focused exclusively on worries related to personal finances measured with three items answered on a 5-point scale from “not at all” to “always.” In the Wu et al. study, on the other hand, economic stress was measured with three yes/no items with a broader focus, incorporating housing and employment stress in addition to “financial status.” It is possible that this broader conceptualization of strain could have diluted and rendered undetectable any gender-specific association existing only between personal financial worry/stress and mental health, especially given the somewhat coarse way in which strain was measured.

A surprisingly strong finding in the present study was the clear gender-specific association between social strain and SWB, with no association found in females but a marked association in males. A gendered effect of loneliness on mental health was found in the Wu et al. study, although the adverse effect appeared to be stronger in females, at least at certain phases of resettlement. Comparing these findings is not straight-forward, however, since two of the social strain items in our study focused on frustration due to loss of status in Swedish society and frustration caused by not being able to use personal competency, both conceptually clearly distinct from loneliness. In a more explorative vein, a potential explanation for the apparent stronger negative effect of social strain in males found in the present study, may be related to traditional gender roles and the value placed on patriarchy in Middle Eastern societies, with men's identity closely linked to work and being able to provide for their family (54, 55). This argument is broadly in line with evidence from Syrian refugees in Lebanon where men frequently reported feeling ashamed to ask for help from aid agencies and often sent women to pick up boxed food (56). However, it is important to not try to fit a highly complex gendered reality into stereotypical and overly simplistic gender models (57). More studies are needed to corroborate this interesting and seemingly robust finding.

The notable reduction in the size of the ORs when going from unadjusted to fully adjusted models for financial strain and social strain across all strata was largely explained by the shared variance between these two variables. That is, the association between financial strain and SWB was markedly reduced upon adding social strain to the model, much more so than when adding the other variables included in the full model. Similarly, the association between social strain and SWB was clearly reduced upon adding financial strain to the model. In fact, for women, there was some statistical evidence of an association between social strain and SWB if financial strain was left out of the full model. This association, however, was completely absent when financial strain was included. Considering the three items comprising each of the two post-migration stressors (see Supplementary Table 1), a possible explanation could be that part of the adverse effects of social strain on SWB, particularly for women, is mediated through financial strain—i.e., social strain leads to financial strain, which then impacts SWB. Furthermore, the reduction in the size of the ORs for competency strain when going from crude to fully adjusted models was strongly linked to the shared variance between competency strain and both financial and social strain, with ORs pushed notably toward the H_0_ value of 1.00 when either or both were controlled for. Disentangling cause and effect between the post-migration stressors and well-being is complicated by the fact that these factors are likely tightly intertwined, mutually and continuously influencing each other. It is possible that including all stressors in the same model masks true associations because of overadjustment bias (58). For example, if competency strain has an adverse effect on SWB that causally goes through social strain (i.e., high competency strain → high social strain → low SWB), then this effect may be “controlled away” when including both stressors in the same model.

The moderate evidence that social support buffered the adverse effects of financial strain on SWB in the present study is broadly in line with another study from Sweden (36), which found that social support moderated the negative effects of discrimination on mental health. Similarly, the modifying role of social support was a main finding and take-home message in the Canadian Refugee Resettlement Project, which found that the positive association between resettlements stress and depression was only evident for refugees lacking personal and social support (37). Both of these studies, however, focused on psychological distress or disorder, and not on positive mental health as the present study did. The importance of social support in refugees may also depend on where the support comes from, as indicated by one study on refugees from Sudan resettled in Australia which found that social support from family and from within the Sudanese community was associated with mental health, but not support from the wider community (59). The absence of statistical evidence for interaction between social support and financial strain when analyses were stratified by gender was likely because the study was underpowered to detect this two-way interaction.

### Strengths and Limitations

The present study has several strengths. First, by approaching mental health with a clear gender perspective, the study mitigates a key limitation in refugee research to date, namely the paucity of gender-specific data and evidence (40, 41). The findings on gender-specific effects of stress in the resettlement phase may be applied in practice to support and promote gender-sensitive resettlement strategies and initiatives, as well as facilitate research focusing on identifying predictors of well-being outcomes. A second strength is the methodological rigorousness of the study, and arguably the focus on positive mental health as opposed to a deficit model of health, given the predominance of the latter approach in current literature. As highlighted in a recent review article (23), one of the limitations of studies on positive mental health in refugee populations relate to weaknesses in methods and design—e.g., the frequent use of convenience samples. In the present study participants were recruited through random sampling from total population registries and the large number of respondents increased the power to detect smaller associations. Furthermore, validated measurement instruments were used for outcome and key predictor variables, and a broad set of relevant contextual factors were included in analysis, potentially reducing confounding noise and spurious associations distorting the main associations of interest.

The study also has important limitations. Given that just above 30% of invited refugees decided to participate in the study, there is a risk that selection bias may have affected results. Supplementary Table 2 shows that there were statistical differences between respondents and non-respondents on several sociodemographic variables. However, as shown in a prior publication (14), weighted and unweighted prevalence estimates for SWB and other mental health parameters were similar in the study population, making it less likely that non-response bias has greatly distorted results. When final regression models in the present study were repeated using weighted analysis (poststratification weights), there was only negligible differences in the statistical evidence behind findings and ORs remained mostly unchanged. Moreover, it is not necessarily straightforward, or recommended, to use weighted analyses when exploring causal effects (60).

Since the study focused exclusively on refugees from Syria resettled in Sweden between 2011 and 2013, the findings may not be generalized to other refugee populations, or even to Syrian refugees coming at different time-periods as the profile of refugee cohorts from Syria may vary between time-periods.

Although the refugee post-migration stress scale (RPMS) used in the present study has been piloted and validated in refugee populations, it is possible that some of the questions in the scale rest too heavily on assumptions and expectations found predominantly in gender-equal societies and less in societies with greater gender separation. For example, a Syrian woman may interpret and answer the question “Feeling excluded or isolated in Swedish society” differently than a Syrian man because of gender role expectations rooted in traditional Syrian society and culture.

The cross-sectional design of the study places clear limits on causal interpretations. This point is particularly relevant in light of the recent findings by Wu et al., which suggest that the effects of post-migration stress on mental health are time-varying (16).

Lastly, no pre-registered study protocol with a detailed analysis plan exists, and analytic choices were partly made with data at hand. The study is therefore somewhat exploratory in nature even if hypotheses were based on previous evidence, and relevant theory and were broadly conceptualized prior to approaching the data.

## Conclusion

Post-resettlement stress related to financial and social hardships appears to have highly gendered effects on SWB in resettled refugees, with a notably stronger adverse effect in men. Policies and interventions aimed at mitigating psychological distress in refugee populations should be sensitive to this finding and accommodate the potentially gendered reality in order to enhance effectiveness. Given the limited research that has been conducted on this topic and the variations in findings, further research with robust methodology (e.g., randomly selected large samples, validated measurements and longitudinal designs) is clearly needed.

## Data Availability Statement

The statistical code is available from the corresponding author. Under Swedish law and ethical approval, individual level data of this kind cannot be publicly available. Individual level data can be made available on reasonable request as long as it is in line with Swedish law and ethical approvals.

## Ethics Statement

The studies involving human participants were reviewed and approved by Swedish Ethical Review Board. The patients/participants provided their written informed consent to participate in this study.

## Author Contributions

NA organized the database, performed the statistical analysis, wrote the statistical analysis, and results section of the manuscript. SM wrote the first draft of the manuscript. All authors contributed to conception, design of the study, manuscript revision, and approved the submitted version.

## Funding

This study was supported by Swedish Research Council for Health, Working Life, and Welfare (Grant Number 2016-07194), the Swedish Ministry of Employment, with additional financial support from the Swedish Red Cross and the Swedish Red Cross University College.

## Conflict of Interest

The authors declare that the research was conducted in the absence of any commercial or financial relationships that could be construed as a potential conflict of interest.

## Publisher's Note

All claims expressed in this article are solely those of the authors and do not necessarily represent those of their affiliated organizations, or those of the publisher, the editors and the reviewers. Any product that may be evaluated in this article, or claim that may be made by its manufacturer, is not guaranteed or endorsed by the publisher.
