# Supplementary material for: Post-migration Stressors and Subjective Well-Being in Adult Syrian Refugees Resettled in Sweden: A Gender Perspective
Source: Front Public Health. 2021 Sep 9;9:717353. doi: 10.3389/fpubh.2021.717353 (PMC8458654; doi:10.3389/fpubh.2021.717353)
Supplement: Supplementary file 1 [file Data_Sheet_1.docx]

| Table 1S The refugee post-migration stress scale (RPMS). Please indicate how frequent you experience each of the following situations in Sweden. Never/Seldom/Sometimes/Often/Very often |
| --- |
| Perceived discrimination |
| Discrimination by Swedish authorities |
| Discrimination in school or at work |
| Feeling disrespected due to my national background |
| People making racist remarks towards me |
| Lack of host-country specific competencies |
| Bothering difficulties communicating in Swedish |
| Difficulties understanding how ordinary life activities in Sweden work (shopping, buying tickets, traveling, etc.) |
| Difficulties understanding documents and forms from authorities |
| Material and economic strain |
| Worry about unstable financial situation |
| Frustration for not being able to support myself financially |
| Worry about debts |
| Loss of home country (heimat) |
| Missing my social life from back home |
| Longing for my home country |
| Missing activities that I used to do before coming to Sweden |
| Family and home country concerns |
| Worry about family members that I am separated from |
| Feeling sad because I am not reunited with family members |
| Social strain |
| Feeling excluded or isolated in the Swedish society |
| Frustration due to loss of status in the Swedish society |
| Frustration because I am not able to make use of my competences in Sweden |
| Family conflicts |
| Distressing conflicts in my family |
| Feeling disrespected in my family |
| Feeling unimportant in my family |
