# Supplementary material for: Post-migration Stressors and Subjective Well-Being in Adult Syrian Refugees Resettled in Sweden: A Gender Perspective
Source: Front Public Health. 2021 Sep 9;9:717353. doi: 10.3389/fpubh.2021.717353 (PMC8458654; doi:10.3389/fpubh.2021.717353)
Supplement: Supplementary file 2 [file Data_Sheet_2.docx]

| Table 2S Descriptive statistics on sample vs. respondents | | | | | | | | | |
| --- | --- | --- | --- | --- | --- | --- | --- | --- | --- |
|  |  |  | Sample (N=4000) | |  | Respondents (n=1215) | |  | Respondents vs non-respondents |
|  |  |  | n | (%) |  | n | (%) |  | χ2 p-value |
| Gender |  |  |  |  |  |  |  |  | 0.52 |
|  | Male |  | 2521 | (63.5) |  | 763 | (62.8) |  |  |
|  | Female |  | 1459 | (36.5) |  | 452 | (37.2) |  |  |
| Age |  |  |  |  |  |  |  |  | <0.01 |
|  | 18-29 |  | 1228 | (30.7) |  | 283 | (23.3) |  |  |
|  | 30-39 |  | 1346 | (33.7) |  | 400 | (32.9) |  |  |
|  | 40-49 |  | 841 | (21.0) |  | 295 | (24.3) |  |  |
|  | ≥50 |  | 585 | (14.6) |  | 237 | (19.5) |  |  |
| Education |  |  |  |  |  |  |  |  | <0.01 |
|  | 0-9yrs |  | 1696 | (44.2) |  | 453 | (38.4) |  |  |
|  | 10-12yrs |  | 893 | (23.3) |  | 255 | (21.6) |  |  |
|  | 13-14yrs |  | 742 | (19.3) |  | 234 | (19.9) |  |  |
|  | ≥15yrs |  | 507 | (13.2) |  | 237 | (20.1) |  |  |
| Civil status |  |  |  |  |  |  |  |  | <0.01 |
|  | Married |  | 2114 | (52.9) |  | 771 | (63.5) |  |  |
|  | Unmarried |  | 1631 | (40.8) |  | 386 | (31.7) |  |  |
|  | Div./wid. |  | 255 | (6.3) |  | 58 | (4.78) |  |  |
| Year immigration |  |  |  |  |  |  |  |  | <0.01 |
|  | 2008-2011 |  | 404 | (10.1) |  | 76 | (6.3) |  |  |
|  | 2012 |  | 1180 | (29.5) |  | 334 | (27.6) |  |  |
|  | 2013 |  | 2416 | (60.4) |  | 802 | (66.1) |  |  |
